# Evaluating cloudcare, a population health management system, in persons with type 1 diabetes: an observational study

**DOI:** 10.1186/s12902-025-01905-4

**Published:** 2025-03-31

**Authors:** Cornelis A. J. van Beers, Sander Last, Pim Dekker, Erwin Birnie, Nico Riegman, Francisca van der Pluijm, Christine Fransman, Henk J. Veeze, Henk-Jan Aanstoot

**Affiliations:** 1 Diabeter Nederland, Center for Pediatric and Adult Diabetes Care and Research, Rotterdam, The Netherlands; 2 Medtronic Trading NL B.V, Eindhoven, The Netherlands; 3 Department of Genetics, University Medical Center Groningen, University of Groningen, Groningen, The Netherlands

**Keywords:** Type 1 diabetes, Population health management, Remote monitoring, Care pathways

## Abstract

**Background:**

Innovations in diabetes technology have consistently improved outcomes of persons with type1 diabetes (PWDs). However, the volumes of data that these technologies yield require different workflows to alleviate healthcare professionals’ (HCPs) workload and prevent losing relevant data in between visits for interpretation and treatment adaptations. CloudCare is a population health management tool that continuously oversees data from groups of individual PWDs, based on remote monitoring, screening and triaging of individual PWDs. This study assesses the effect of CloudCare on treatment satisfaction of PWDs, HCPs’ workload and glycemic control of PWDs.

**Methods:**

We evaluated the 6-month follow-up outcomes as part of an ongoing prospective cohort study analyzing the effect of CloudCare. Adult PWDs diagnosed > 6 months before inclusion were enrolled. The primary outcome was the change in PWD treatment satisfaction (DTSQc). Secondary outcomes included the number and type of contacts between HCPs and PWDs, diabetes-related distress (PAID-5), and glycemic control.

**Results:**

In September 2024, 175 participants had baseline data available, with a median age of 29.9 years and a median diabetes duration of 17 years. Differences between baseline and 6 months could be calculated for 119 participants. After 6 months follow-up, the median increase in PWDs’ treatment satisfaction (DTSQc) was + 6.0 (IQR 2–11; *p* < 0.001). The number of face-to-face contacts per PWD per 3 months decreased from 0.85 at baseline to 0.34 (*p* < 0.001) at 6 months. Diabetes-related distress was significantly decreased at 3 months (*p* < 0.001) and at 6 months (*p* = 0.034), compared with baseline. Glucometrics did not significantly change, with a TIR of 79% at baseline and 78% after 6 months (*p* = 0.39), and a mean glucose management indicator (GMI) of 50 mmol/mol (6.7%) at all timepoints.

**Conclusions:**

In adult PWDs with good glycemic control, CloudCare decreases workload for HCPs, while increasing PWDs’ treatment satisfaction and maintaining excellent glycemic control during 6 months, showing this concept can be applied in modern diabetes care with high density data availability.

**Trial registration:**

Clinicaltrials.gov identifier: NCT05431140; registration date 21-6-2023.

## Introduction

The development of diabetes technology has significantly improved the glycemic control and quality of life of persons with type 1 diabetes (PWD) [1]. Despite these innovations many PWDs do not reach glycemic targets [2], exposing them to an increased risk of diabetes-related morbidity and mortality [3]. One opportunity for further improvement of outcomes is translating the large volumes of data generated by diabetes technology (e.g. continuous glucose monitor [CGM] data) into patterns and trends in order to enable self-management adaptations. The Ambulatory Glucose Profile (AGP) provides a visual representation of the glycemic control of a PWD, which can be reviewed by the healthcare professional (HCP) together with the PWD. This helps to define individual treatment targets and make adjustments to self-management [4].

Despite AGPs, interpreting and translating the data into relevant actions is time consuming and can be overwhelming for both HCPs and PWDs [5]. Considering the projected shortage of medical personnel and increase in healthcare needs [6], new solutions are needed to prevent overflow of the healthcare system. Current approaches in analyzing glucose data are based on sequential (i.e. individual-by-individual) assessments of PWDs. This strategy is time consuming and inefficient, since not only the PWDs at risk of acute (severe hypoglycemia, diabetic keto-acidosis) and long-term (microvascular and macrovascular) complications [7, 8, 9] are analyzed, but also the PWDs who do not need self-management support at a specific moment in time. Also, much information in between assessments is lost [10]. That is why there is a need for systems that continuously oversee larger groups of PWDs simultaneously, which is an integral concept of population health management (PHM). PHM enables improvement of health outcomes and quality of life of a defined group of individuals through improved care coordination and patient engagement, and more efficient use of resources [11]. Current data suggests that these PHM strategies can potentially improve outcomes and decrease HCP workload [12, 13, 14]. However, operationalization of PHM in practice is currently limited [15].

Diabeter, a Dutch center for pediatric and adult diabetes care and research, has developed a CE-marked PHM tool called CloudCare [16], a remote monitoring application and triaging service which integrates into clinical workflows. The (brand-agnostic) CloudCare application daily gathers glucose data from multiple PWDs simultaneously (i.e. the whole population of a certain clinic or HCP) and translates it into internationally defined glycemic parameters, such as Time In targeted glucose Range (TIR) and Glucose Management Indicator (GMI) [17], which are associated with relevant clinical outcomes [7, 8, 9]. Based on a triaging protocol, PWDs at risk of acute and long-term complications can be timely identified. This enables HCPs to proactively assess (glucose) data and discuss self-management adaptations accordingly. On the other hand, if glucose values do not deviate, detailed analysis of an AGP by the HCP is not necessary, theoretically saving scarce healthcare resources. In this observational study we assessed if there are associations between using CloudCare in our care pathway and PWDs’ treatment satisfaction, the workload of HCPs (based on number and type of contacts between PWD and HCP), glycemic control and diabetes-related distress.

## Methods

### CloudCare

CloudCare is a clinician-developed PHM tool and is registered as a Medical Devices Regulation (MDR) class IIa medical device. Using Cloudcare, a dedicated team of HCPs (the ‘CloudCare team’) is able to remotely monitor PWDs and identify those whose glucose values deviate. CloudCare daily collects glucose data via different data sources (e.g. Carelink, Glooko, Libreview) and translates these glucose values into clinically relevant glycemic parameters [16]. These glucometrics are then converted to a dashboard showing the different glycemic parameters of each PWD and colored for level in a way intuitive to both PWDs and HCPs: the heatmap. Five glucometric parameters are color-coded: TIR, high blood glucose index (HBGI), low blood glucose index (LBGI), GMI, and glucose standard deviation. These parameters and their color coding are adaptable based on clinical preferences. Diabeter has developed a triage protocol applying these color-coded indicators and international guidelines. During onboarding on the CloudCare pathway, the HCP and PWD align on a new planned (reduced) visit sequence, taking into account preferences of the PWD. Based on the status of the indicators and the PWDs’ contextual data points, PWDs are triaged and those who have deteriorated with respect to certain values are forwarded to the care team. At the HCPs’ discretion actions can be initiated to contact the PWD. The filter settings can be adjusted. Both the PWD (via an application) and the HCPs have access to the same heatmap, promoting efficient (remote) consultations regarding treatment adaptations. Using CloudCare allows the HCP team to transform its care model and drive hybrid, personalized and data driven care pathways.

### Study population

Included were PWDs for whom CloudCare is part of their (new) standard of care. The study population comprised PWDs diagnosed with type 1 diabetes > 6 months before inclusion (by pediatrician or internist specialized in endocrinology and in accordance with internationally accepted guidelines), aged between 16 and 75 years and treated with insulin with or without metformin (either via multiple daily injections [MDI] or continuous subcutaneous insulin infusion [CSII]), using intermittent-scanning CGM (is-CGM) or real-time CGM (rt-CGM) for > 3 months without CloudCare, but with onboarding for CloudCare planned as part of their standard of care, lasting for at least 6 months. While no HbA1c limit was set for inclusion, in our clinical practice CloudCare was initially implemented in PWDs with good glycemic control (time in range of > 70%) to minimize safety risks. Exclusion criteria included the use of glucose lowering therapy other than insulin or metformin and any known factors, conditions or diseases that might interfere with study conduct or interpretation of the results, determined at the discretion of the treating HCP. Otherwise, all PWDs who registered for the study and provided informed consent were included.

### Study design

**Fig. 1:**
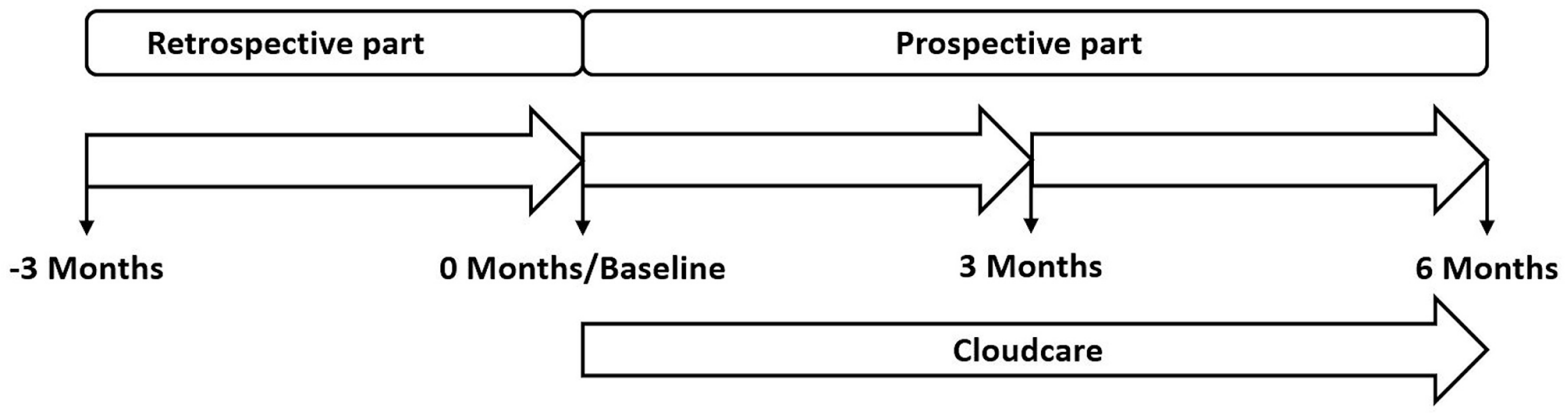
Study outline

We performed a single-center, prospective cohort study evaluating the effect of the integration of CloudCare into existing care pathways on PWDs’ treatment satisfaction, the number and type of contacts between HCPs and PWDs, diabetes-related distress, and glycemic control (Clinicaltrials.gov/study/NCT05431140; registration date 21-6-2023). Participants were enrolled in one of the five locations of Diabeter, the Netherlands, which were managed as one study site. Eligible PWDs were invited by email to participate in the study. Interested potential participants received information describing the study, the type of data that would be collected and the intended analyses. After providing (digital) informed consent, eligible PWDs were enrolled. The study consisted of two periods: a retrospective, pre-baseline period of three months in which data was retrieved from PWDs’ electronic health records (EHRs), including device data, followed by prospective observational periods of three and six months after introduction of CloudCare (Fig. 1). The retrospective period served as the control period for the prospective periods. The study was conducted in full compliance with the study protocol and the principles of the Declaration of Helsinki (www.wma.net). The study was exempt from further approval procedures as participants were not subjected to any interventions, actions or restrictions and are followed in regular care (Medical Research Ethics Committee of Erasmus University Medical Centre [MEC-2019-0790], Rotterdam, The Netherlands). To improve the validity of this observational study by analyzing participants according to their initial treatment, even if they stop treatment, the data were analyzed according to the intention-to-treat (ITT) approach.

### Outcomes

The primary outcome was the change in treatment satisfaction score at 6 months from baseline, assessed with the change version of the Diabetes Treatment Satisfaction Questionnaire (DTSQc) [18]. According to the official scoring model, the total treatment satisfaction score is defined as the unweighted sum score of items 1 and 4—8 [19]. Items are scored on 7-point Likert scales (-3: much less; +3: much more). Secondary outcomes were: the number and type of contacts between PWDs and HCPs per PWD per three months, diabetes-related distress using the Problem Areas in Diabetes Scale-5 questionnaire (PAID-5; a higher PAID-5 score indicates more diabetes-related distress, with a score of ≥ 8 as a threshold for high [clinically relevant] distress) [20], and glycemic parameters (HbA1c, glucose management indicator [GMI], Time In targeted glucose Range [TIR: 70–180 mg/dL], Time Above targeted glucose Range [TAR: >180 mg/dL] and Time Below targeted glucose Range [TBR: <70 mg/dL]). Outcomes were evaluated at baseline, 3 months and 6 months.

### Data collection

Participants’ clinical data were retrieved from their EHRs and glucose data from their medical devices used for their type 1 diabetes treatment. Both were entered in the study database (Castor Electronic Data Capture [EDC] platform [21]). Questionnaire data were collected through the study database platform and results were automatically uploaded into the study database. For the pre-baseline 3-month period, participants’ baseline characteristics and therapy details were collected. At baseline, data were collected on demographic and anthropomorphic characteristics, therapy details, laboratory HbA1c, glucometrics (GMI, TIR, TAR, TBR) and relevant medical history. Additionally, participants completed the PAID-5 and data on the number and type of contact moments between PWD and HCP (i.e. face-to-face, telephone, videocall, e-mail or letter) were collected. At 3 and 6 months the same parameters were collected. However, the DTSQ license does not allow DTSQc scores to be assessed between 3 months and baseline. Consequently mean DTSQc score was only assessed between 6 months and baseline. DTSQc and PAID-5 questionnaires were validated and available in Dutch. Glucometrics (GMI, TIR, TAR, TBR) were based on the 3 weeks of CGM glucose values prior to the 3 time points (baseline, 3 months and 6 months).

### Statistics

To be able to reject the null hypothesis (mean DTSQc score at 6 months = 0) with sufficient statistical power (90%), assuming that the mean intrapersonal difference in DTSQc score is + 2.0 at 6 months follow-up, 116 PWDs were calculated to be needed for the analysis. To adjust for 45% drop-out and non-response, it was calculated that (at least) 194 PWDs needed to be enrolled in the study. Continuous data with normal distribution were summarized as means with SD and 95%CI, continuous data with skewed distribution as medians with interquartile range (IQR, Q1-Q3) and nominal and ordinal data as n (%). The skewness of the distributions was evaluated using the Shapiro-Wilk test. For analysis of the primary outcome, a *p*-value (two-sided) < 0.025 (Bonferroni-adjusted) was considered statistically significant. For all other analyses, a *p*-value (two-sided) < 0.05 was considered statistically significant.

Changes in DTSQc score were tested between 6 months and baseline using one sample Wilcoxon signed test. The changes in mean HbA1c, GMI, TIR, TAR and TBR between 3 months and baseline months and between 6 months and baseline were evaluated with repeated measurements analysis (linear mixed model; dependent variable, glucometric parameter; covariable, time; covariance structure, unstructured), as were changes in PAID-5 score. Numbers of contacts with HCP by type of contact (total, face-to-face, other than face-to-face) between 3 months and baseline and between 6 months and baseline were tested with generalized linear mixed model random effects negative binomial regression analysis. These analyses are capable of analyzing trends of outcomes over time, even when outcome data are partially incomplete. Loss to follow-up was only addressed as part of the repeated measurements analyses (linear mixed model). No imputation occurred. Numbers of PWDs may vary over time due to the longitudinal design (incomplete follow-up) and missing values (e.g. questionnaire non-response).

## Results

The increase in DTSQc score was higher than the anticipated + 2 points, which prompted us to analyze the results of the first 119 patients who completed 6 months follow-up. In September 2024, 180 PWDs had provided informed consent of which 5 refrained from starting the study.

Table 1 shows the baseline characteristics of the participants. Median (IQR) age was 29.9 (24.6–42.0) years, 62% were female, median (IQR) diabetes duration was 17 (11–26) years and median (IQR) TIR was 79 (73–84). Most participants (93%) used an insulin pump of whom 87% were using the Minimed 780G AID system, 3% the Minimed 670G system, 2% the Tandem Slim X2 system and 2% another system. 7% of participants used MDI + CGM.

**Table 1:** Baseline characteristics

Characteristic	*n*	Median (IQR) unless specified otherwise
Age, years	175	29.9 (24.6–42.0)
Female, n (%)	175	108 (61.7)
Diabetes duration, years	175	17 (11–26)
Lab HbA1c	127	
mmol/mol %		48.0 (44.0–51.9) 6.5 (6.2–6.9)
GMI, mean (SD)	149^a^	
mmol/mol %		50.1 (3.2) 6.7 (0.3)
Glucometrics	154^a^	
TIR (70–180 mg/dL) TAR (> 180 mg/dL) TBR (< 70 mg/dL		79 (73–84) 19 (13–25) 2 (1–3)
Current insulin therapy, n(%)	173^b^	
MDI (FGM) Pump Minimed 670G Minimed 780G Tandem Slim X2 Other		12 (7) 161 (93) 5 (3) 150 (87) 3 (2) 3 (2)

^a^ Glucometrics were not available for *n* = 19 participants due to lack of data availability around the visit dates. GMI data (calculated for ≥ 14 days) are different from TIT/TBR/TAR data [22]

^b^ For *n* = 2 participants it was not clear if they were on MDI or on pump as they were registered for both

FGM, flash glucose monitoring; GMI, glucose management indicator; HbA1c, glycated hemoglobin A1c; IQR, interquartile range; MDI, multiple daily insulin injections; SD, standard deviation; TAR, time above target glucose range; TBR, time below target glucose range; TIR, time in target glucose range

**Fig. 2:**
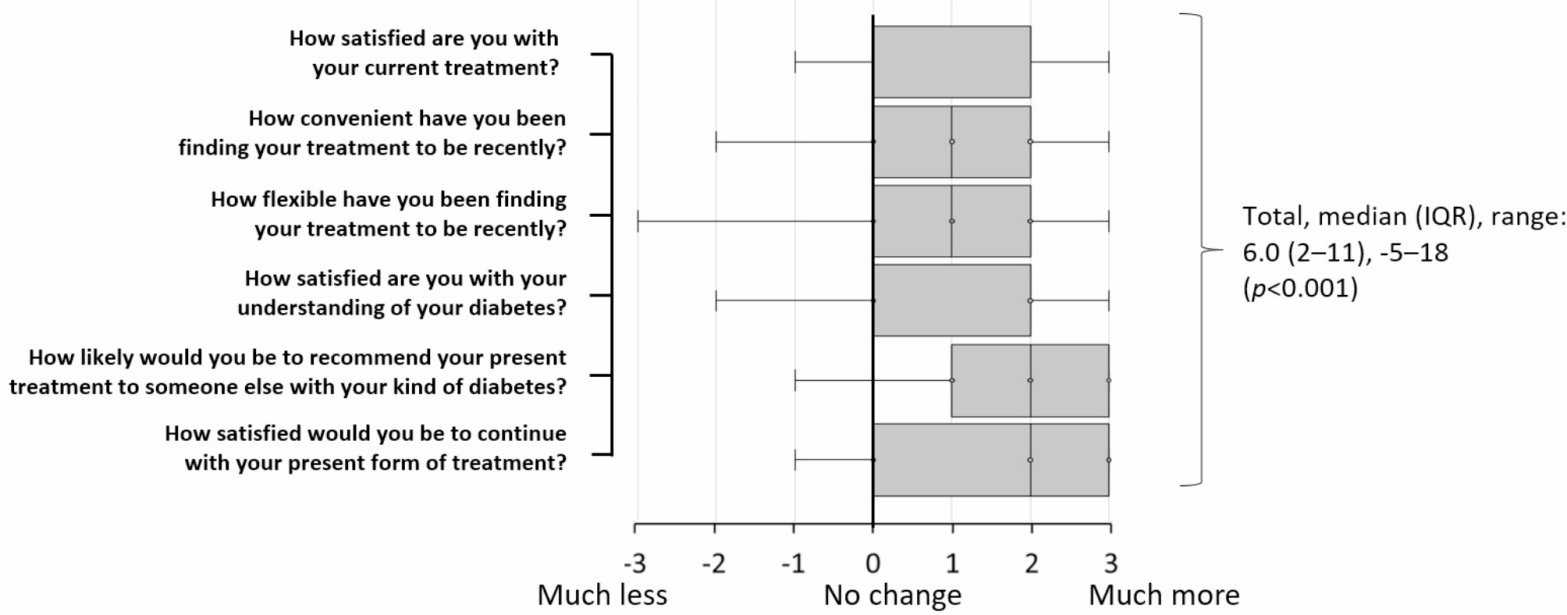
Box and Whisker plot (median, IQR and range) of change in DTSQc score between baseline and 6 months (-3 to + 3 per item; *n* = 119). DTSQc, Change version of the Diabetes Treatment Satisfaction Questionnaire; IQR, interquartile range

Treatment satisfaction (DTSQc) increased significantly between baseline and 6 months (Fig. 2).

**Fig. 3:**
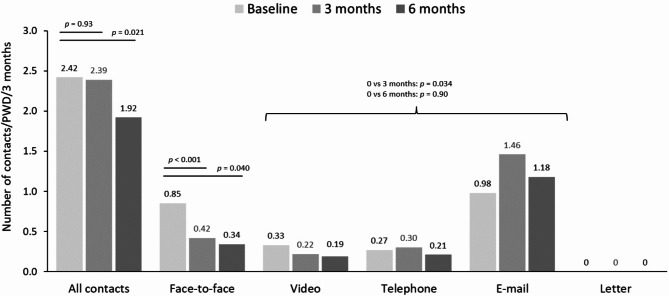
Contacts between PWDs and HCPs (baseline, *n* = 172; 3 months, *n* = 134; 6 months, *n* = 119)

The number of face-to-face contacts significantly decreased from 0.85 per PWD per 3 months at baseline to 0.34 per PWD per 3 months at 6 months. Numbers of video, telephone and e-mail contacts changed significantly after 3 months, mainly due to number of e-mail contacts. However, after 6 months the difference with baseline was no longer significant (Fig. 3). Letters were not used for communication during the study period.

Diabetes-related distress (PAID-5) decreased significantly from baseline to 3 months. Although median PAID-5 at 6 months was similar to baseline, individual changes between 6 months and baseline still showed a significant decrease (Table 2). At baseline, glucose control parameters GMI (estimated HbA1c), TIR, TAR and TBR were 50 mmol/mol (6.7%), 79%, 19% and 2%, respectively, and did not significantly change between baseline, 3 months and 6 months (Table 2). There were no diabetes-related and/or non-diabetes-related hospital admissions during the study period.

**Table 2:** PAID-5, GMI, TIR, TAR and TBR at baseline, 3 months and 6 months (median, IQR and range)

	Baseline	3 months	*P* ^a^	6 months	*P* ^b^
PAID-5, score in points, median (IQR)	*n* = 161 5.0 (3.0–7.5)	*n* = 139 4.0 (2.0–7.0)	**< 0.001**	*n* = 119 5.0 (2.0–7.0)	**0.014**
GMI, mmol/mol, mean (SD)	*n* = 149 50 (3)	*n* = 161 50 (3)	0.34	*n* = 142 50 (4)	0.45
TIR, %, median (IQR), range	*n* = 154 79 (73–84), 54–98	*n* = 162 79 (73–84), 53–97	0.56	*n* = 145 78 (74–84), 47–98	0.39
TAR, %, median (IQR), range	*n* = 154 19 (13–25), 1–45	*n* = 162 20 (14–26), 1–45	0.32	*n* = 145 19 (14–24), 1–51	0.29
TBR, %, median (IQR), range	*n* = 154 2 (1–3), 0–25	*n* = 162 1 (1–2), 0–8	0.45	*n* = 145 1 (1–3), 0–8	0.53

^a^ Change between 3 months and baseline, ^b^ change between 6 months and baseline

IQR, interquartile range; GMI, glucose management indicator; PAID, Problem Areas in Diabetes Scale 5 questionnaire; TAR, time above target glucose range; TBR, time below target glucose range; TIR, time in target glucose range

## Discussion

In this study we show that use of CloudCare resulted in increased PWD treatment satisfaction (DTSQc) after 6 months. Also, the overall number of contacts decreased significantly after 6 months, due to a significant decrease in face-to-face contacts. Although not formally tested, we hypothesize that this may lead to decreased HCP workload and decreased diabetes-related burden. The latter may also partly explain the increase in PWD treatment satisfaction, since this care model minimizes unnecessary in-person or video visits while still providing a safety net when needed. Additionally, after 3 months increases in e-mail contacts and telephone consults were observed. This may be linked to communication related to PWD-onboarding in the CloudCare program. Although not within the scope of this analysis, the email contacts generally fall into three categories: clinical concerns (from both HCPs and PWDs), administrative inquiries (e.g., travel documents or driver’s license certificates), and technical questions. Diabetes-related distress (PAID-5) remained low during the course of the study. Glycemic parameters (GMI, TIR, TAR and TBR) were well above recommended target levels [22] at baseline and did not significantly change throughout the study. We do not consider the numerical decrease of 1% for TIR clinically relevant, with TIR well above target [22].

The ability to access glucose data remotely enables development of new models of type 1 diabetes care [23], which is needed for further improvement of outcomes [2] and the projected scarcity of healthcare resources [6]. However, development of these models is hampered by the current lack of tools and solutions that make large volumes of data actionable for HCPs, risking overwhelming of healthcare systems. The pediatric 4T study is one of the few studies that highlights the unique challenges that clinicians and health systems face when attempting to incorporate a PHM system into clinical care [5]. The 4T study resulted in the Timely Intervention for Diabetes Excellence (TIDE) platform, an open-source platform generating a variety of CGM metrics and identifying PWDs whose metrics should be reviewed, based on flags and alerts. Using the TIDE dashboard, individual HCPs can review up to 28 PWDs per week. However, the authors stated that, despite the advantages to PWDs, the 4T program adds workload to HCPs. This underlines the importance of adapting and changing methods of work using data-analysis technologies.

CloudCare uses elements of population health management which allow a redefinition of workflow for HCPs with the intention to reduce HCPs’ workload. Instead of HCPs retrospectively reviewing glucose data of each individual PWD, usually only before planned consultations, CloudCare daily monitors glucose data for many PWDs simultaneously and continuously. This process is guarded by a dedicated CloudCare team who will alert treating HCPs of any PWDs needing attention.

In addition to enabling healthcare organizations to address staffing challenges, this workflow could also optimize the use of the available data and change clinical care pathways by shifting the focus to those who need extra attention on diabetes self-management, allowing a PWD-centered disease management approach such as outlined in the Chronic Care Model [24]. Compared with traditional care pathways, with evaluation of data at the level of the individual, CloudCare requires HCPs to accept, work and be educated in a model where an automated algorithm oversees groups of PWDs (population management) and identifies those that need review or further attention. From the PWDs’ perspective, innovations in diabetes technology have already improved PWDs’ autonomy to manage their disease, leading to decreased disease burden and improved quality of life [1, 13, 25, 26, 27]. The corona virus disease pandemic showed that remote monitoring and telemedicine have a place in diabetes care. Although these developments cannot entirely replace face-to-face consultation [26, 28, 29], platforms like CloudCare potentially enable a shift from standard multiple face-to-face visits per year towards data-driven remote consultations in between annual face-to-face evaluation visits or blended models of care.

Some limitations need to be addressed. Firstly, our study is a relatively small proof-of-concept study and follow-up of 6 months is relatively short. Secondly, participants in this study had excellent glycemic control, because in our clinical practice CloudCare was initially implemented in PWDs with good glycemic control to minimize safety risks. In the near future we will start using CloudCare in PWDs with less optimal glycemic control. In future studies we will examine the effect of CloudCare in this more heterogenous population. Thirdly, despite the significant increase of treatment satisfaction, the sample size was relatively small and subgroup analysis was impossible. Loss to follow-up was relatively high. This did not hamper the analyses however, as post-hoc power was > 99%. Finally, it is unclear if the results can be extrapolated to the pediatric population.

### Future analyses

To assess to what degree the results are generalizable, the CloudCare study is being continued and 12-months results are underway. The study has also been extended to a multi-center study, including sites from other European countries sites and also with younger participants (children). With more participants included in the study, future analyses will also be able to assess the impact for different subgroups (e.g. sex, age, age at diabetes onset, diabetes duration, previous glucose control, socioeconomic status, complications/comorbidity) and investigate potential associations between treatment satisfaction and number/type of contacts/visits. At the other side of the age spectrum, it will also be interesting to assess how older participants are doing as they tend to have less access to computers, smartphones etc. and also have lower digital literacy [30, 31, 32]. Furthermore, as evidence shows the advantages of is-CGM and rt-CGM in type 2 diabetes, the CloudCare concept is likely to also be applicable to people with type 2 diabetes, regardless of insulin use and despite the even greater challenges to use data of this group appropriately [33]. A higher socioeconomic status has also been associated with better glucose control, educational level and adherence to sensor and AID-automode use [34]. The effect of CloudCare on HCP satisfaction will be assessed, as well as costs and time spent by HCP per PWD, to see if team resources will be levelled by using CloudCare. Ferstad et al. reported that remote review of PWDs with CGM reduced time spent by HCPs while simultaneously improving TIR, thus improving population-level management of type 1 diabetes [13]. It will also be interesting to investigate the effect of CloudCare on the self-management and glucose control of PWDs who are not on AID systems, but on MDI + CGM or sensor-augmented pump treatment [35]. Finally, longer-duration studies will confirm if beneficial effects of CloudCare are sustainable [35, 36].

## Conclusion

Application of CloudCare in this cohort decreases the workload for healthcare professionals, while increasing PWDs treatment satisfaction and maintaining excellent glycemic control. CloudCare therefore has the potential to change care pathways, use data from diabetes devices constantly and more appropriately and help to address the increasing shortage in healthcare resources.

## Data Availability

Data can be provided upon reasonable request.

## Abbreviations

AGP: Ambulatory glucose profile
CGM: Continuous glucose monitoring
CSII: Continuous subcutaneous insulin infusion
DTSQ: Diabetes treatment satisfaction questionnaire
EDC: Electronic data capture
EHRs: Electronic health records
GMI: Glucose management indicator
HbA1c: Glycated hemoglobin A1c
HCP: Healthcare professional
IQR: Interquartile range
is-CGM: Intermittent scanning CGM
MDI: Multiple daily insulin injections
MDR: Medical devices regulation
PAID-5: Problem areas in diabetes scale-5
PHM: Population health management
PWD: Person with type 1 diabetes
rt-CGM: Real-time CGM
SD: Standard deviation
TAR: Time above targeted glucose range
TBR: Time below targeted glucose range
TIDE: Timely Intervention for Diabetes Excellence
TIR: Time in targeted glucose range
